# Visualization of Cellulose Structures with Cesium Labeling and Cryo‐STEM

**DOI:** 10.1002/smll.202500351

**Published:** 2025-05-02

**Authors:** Daniel Knez, Patrick Petschacher, Helmar Wiltsche, Jean‐Luc Putaux, Yoshiharu Nishiyama, Yu Ogawa, Gerald Kothleitner, Tiina Nypelö, Stefan Spirk

**Affiliations:** ^1^ Institute of Electron Microscopy and Nanoanalysis Graz University of Technology Graz 8010 Austria; ^2^ Institute of Bioproducts and Paper Technology Graz University of Technology Graz 8010 Austria; ^3^ Institute of Analytical Chemistry and Food Chemistry Graz University of Technology Graz 8010 Austria; ^4^ Univ. Grenoble Alpes, CNRS CERMAV Grenoble 38000 France; ^5^ Graz Center for Electron Microscopy Graz 8010 Austria; ^6^ Department of Chemistry and Chemical Engineering Chalmers University of Technology Gothenburg 41296 Sweden; ^7^ Department of Bioproducts and Biosystems Aalto University Aalto 00076 Finland

**Keywords:** cellulose nanocrystals, cellulose ultrastructure, cesium labeling, cryo‐scanning transmission electron microscopy

## Abstract

Cellulose, a pivotal component of plant cell walls, is a widely studied biologically derived material with vast potential for numerous applications. However, visualizing the arrangement of individual cellulose molecules within hierarchical structures with electron microscopy has proven challenging due to the material's low contrast and high beam sensitivity. In this study, a novel approach is introduced that combines labeling of cellulose functional groups with high‐contrast cesium counter cations (Cs^+^) in conjunction with atomic resolution scanning transmission electron microscopy (STEM) in annular dark‐field (ADF) mode at cryogenic temperatures. This technique allows for the identification of individual sulfate groups attached to cellulose chains within cellulose nanocrystal hierarchies at Ångström resolution. Systematic comparison of experimentally obtained interatomic Cs^+^ distances with simulations potentially enables the localization of the labeled functional groups at the macromolecular level. The method has the potential to elucidate the polymer chain arrangements in nanoscale soft materials.

## Graphical abstract

Cryogenic ADF‐STEM combined with Cs⁺ labelling of sulfate groups enables direct visualization of individual functional groups within cellulose nanocrystal hierarchies at Ångström resolution. Comparison of experimental Cs⁺ positions with simulations allows localization of labeled sites along glucose chains, advancing structural analysis of beam‐sensitive soft materials at the macromolecular level.

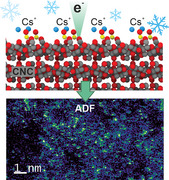

## 1 Introduction

Cellulose occurs in plants as long chains with a degree of polymerization up to ten thousand units, which are organized in supramolecular fibrillar structures. As a consequence of biosynthesis, the cellulose chains in the elementary fibrils are arranged in a mostly crystalline fashion, with short‐range longitudinal dislocations (2–4 nm) disrupting crystallinity.^[^ ^1^, ^2^, ^3^ ^]^ For native cellulose allomorphs I_α_ and I_β_, the average positioning of the chains in the crystallographic unit cell has been determined by X‐ray diffraction techniques.^[^ ^4^, ^5^ ^]^ However, elucidation of the arrangement of the cellulose chain in supramolecular structures requires direct visualization. A prominent example is the current discussion on the number of cellulose chains building up a cellulose elementary fibril. The 6 × 6 chain model has been popular among the plant cell wall community^[^ ^6^ ^]^ but has been challenged by computational scientists and experimentalists in the past years, introducing a wide range of alternative models comprising fewer chains (18, 24, and combinations thereof).^[^ ^7^, ^8^, ^9^, ^10^ ^]^ Recently, the debate about the number of chains in an elementary fibril, 18 or 24, has intensified, and most currently, the occurrence of a mixture of both has also been proposed.^[^ ^9^, ^11^, ^12^ ^]^ Another open question concerns the cross‐sectional shape of the fibril. While both diamond and rectangular cross sections have been proposed, the real morphology is still largely unclear.^[^ ^13^, ^14^ ^]^ The identification of individual cellulose chains has been challenging even with atomic force microscopy and atomic resolution electron microscopy, which in theory, are capable of delivering this information. However, direct visualization of cellulose chains derived from plants with atomic force microscopy is limited by the tip radius needed for imaging the rather small cellulose particles derived from plants. Visualization was demonstrated some years ago, e.g., for crystalline cellulose extracted from valonia.^[^ ^15^ ^]^ Electron microscopy is limited in resolution by the high susceptibility of cellulose to beam damage concomitant with the low electron contrast of light elements (C, O, H) in carbohydrates.^[^ ^16^ ^]^ These problems have partly been addressed by sputtering, staining with uranyl acetate, and low voltage imaging, and indeed progress has been made regarding the visualization of nanoscale cellulose features.^[^ ^17^, ^18^, ^19^ ^]^ However, the atomic scale visualization of cellulose chains in plant cellulose microfibrils has remained inaccessible.

The crystalline regions of the elementary fibrils can be liberated using mineral acids and resulting in cellulose hierarchies called cellulose nanocrystals (CNCs). CNCs have been revolutionary for enabling cellulose materials to enter nanotechnology and partly to access information regarding the fundamental structure of cellulose.^[^ ^20^, ^21^ ^]^ CNCs produced using sulfuric acid hydrolysis are surface‐functionalized with sulfate groups and are the most common type of CNCs. The strategy that we highlight toward visualizing cellulose chains is to exploit the sulfate groups as anchors, which allows us to equip the CNCs with a heavy element ion via a cation exchange. We have established and demonstrated the exchange of the sodium counterion to alkali metal ions on the sulfated CNCs.^[^ ^22^ ^]^ Here, for the visualization, cesium ions are of particular interest as they feature a high atomic number (*Z* = 55), providing large contrast in transmission electron microscopy, potentially reducing exposure time in STEM acquisition and thereby reducing beam damage.

**Figure 1:**
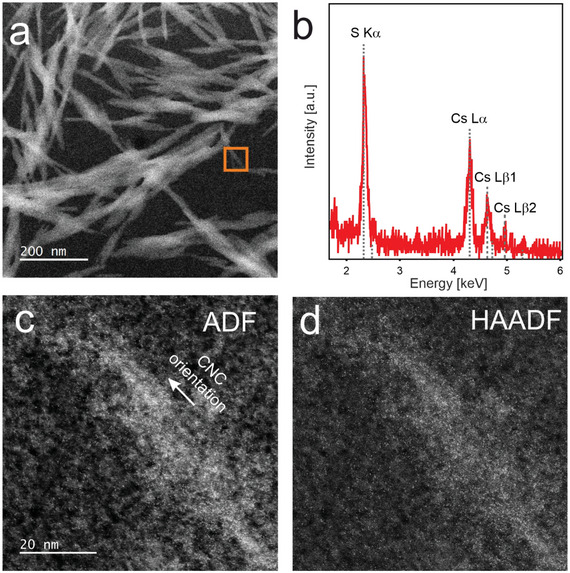
a) ADF Overview image of bundled and aggregated CNCs on a supporting thin (≈2 nm) amorphous carbon layer. b) EDX analysis showing the presence of S and Cs. c) ADF and d) simultaneously acquired HAADF image showing a CNC decorated with bright spots indicating the position of Cs^+^ ions on a thin amorphous carbon support.

The cation exchange itself yields Cs^+^‐CNC materials where ≈86% of the sulfate groups carry a Cs^+^ ion, (and 6% Na^+^ and 8% H^+^), with a total estimated surface coverage of 15% based on elemental composition (Table S1, Supporting Information).^[^ ^23^ ^]^ Importantly, our investigation revealed that the intrinsic crystallinity (cellulose I with minimal traces of cellulose II) of CNCs remained unaffected by the introduction of Cs^+^ ions, with no observed crystallization induced by cesium incorporation (Figure S1, Supporting Information). Overview STEM ADF images of the Cs^+^‐CNCs are presented in **Figure** **1**a and reveal that the individual crystallites are forming bundles with lengths and widths of ≈50–200 and 10–50 nm, respectively. These bundles occur in larger aggregates distributed over the whole amorphous carbon support film. Note that the CNC bundles are oriented randomly due to the drop cast preparation of the CNCs dissolved in water, while the CNCs in each bundle are aligned parallel. STEM‐EDX elemental analysis obtained from larger aggregates of CNCs exhibits clear signals for S (K_α_) and Cs (L_α_, L_β1_, L_β2_), evidencing the presence of Cs^+^ labeled CNCs (Figure 1b).

The positions and distribution of individual Cs^+^ ions were precisely identified using aberration‐corrected STEM at cryogenic temperatures with annular dark‐field (ADF) and high‐angle ADF (HAADF) signals. The resulting images depicting a strand of CNCs are showcased in Figure 1c. The HAADF signal is particularly well‐suited for imaging heavier elements due to its Z^1.7^ proportionality, where Z represents the atomic number.^[^ ^24^ ^]^ As a result, Cs^+^ ions appear as bright spots superimposed over a thickness‐dependent grey background, primarily derived from lighter elements such as carbon, hydrogen, and oxygen, constituting the carbon support of the TEM grid and the CNCs positioned on top.^[^ ^25^ ^]^

**Figure 2:**
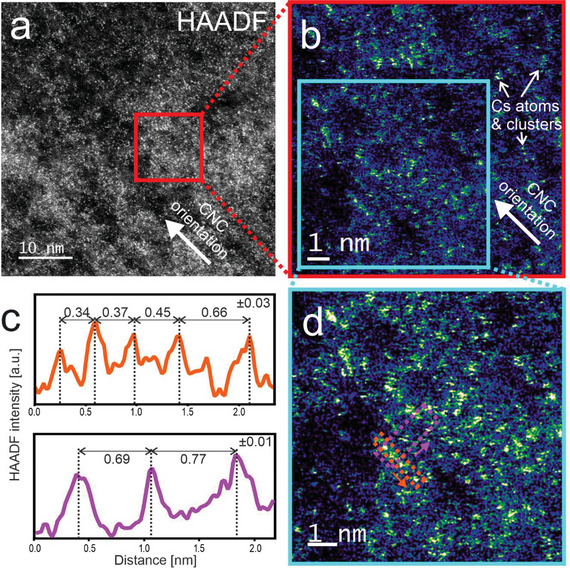
Analysis of the characteristic distances of the Cs^+^ ions on the CNC. a) high resolution HAADF image of a region with a bundle of CNCs. The main orientation of the CNCs long axis [001] is indicated with an arrow. b) Magnification of a region in (a) (marked red) 3 × 3 average filtered for better visibility of the Cs^+^ ions. Selected individual Cs^+^ ions and clusters are denoted by arrows. c) Intensity profiles parallel and perpendicular to the CNC long axis and their measured repetitive distances extracted from the regions highlighted in d). Peak positions are determined by Gaussian fitting, and error values of ± 0.03 and ± 0.01 nm are estimated for the distances measured parallel and perpendicular to the long axis, respectively. Figure (d) is a magnification of the selected region in (b) (marked cyan).

**Figure** **2**a showcases a higher magnification micrograph of the region enclosed by an orange rectangle in Figure 1a. Despite some beam damage indicated by dark “holes” in the image, strands of the sulphated cellulose molecules on the CNC can be observed where individual Cs^+^ ions are visible as bright dots with diameters of ≈100–150 pm (measured as full width at half maximum). The blurry or non‐spherical appearance of some of the single Cs^+^ ions most likely results from their occasional movement while being probed by the electron beam. Such variations in the intensity and feature size are to a large extent, attributed to atomic movement induced by impinging electrons.^[^ ^26^, ^27^ ^]^ Due to the extremely high susceptibility of cellulose to electron irradiation, significant damage effects like atomic displacements and structural degradation are observed as well. Despite such damaging effects in several regions, a distinctive pattern emerges, revealing elongated alignments of Cs^+^ ions extending over several nanometers, closely mirroring the primary orientation of the CNCs (as illustrated in Figure 2b). This distinctive arrangement strongly suggests that the Cs^+^ ions are positioned on the faces of the CNC crystallites, where their bonding to surface sulfate groups results in charge neutralization. Along the lines, where individual Cs^+^ ions are resolved, interatomic distance values between 3 to 7 Å were determined, dependent on direction relative to the crystallite, as determined from the intensity profiles in Figure 2c, which correspond to the regions highlighted with matching colours in the micrograph in (b). These distinctive “Cs^+^ lines” are found to be aligned in a parallel fashion (as depicted in Figure 2b), with at least five discernible lines within the imaged structure. The distance between these parallel lines is ≈6 to 7 Å. Moreover, a comprehensive analysis yields a total CNC diameter of ≈2.5–3.5 nm at this specific location, falling within the expected size range for 18 to 24‐chain models. A magnified and filtered version of the image, better visualizing individual CNC crystals, is provided in the SI. This structural insight provides valuable information about the spatial arrangement of the Cs^+^ ions within the CNC structure and their interaction with the sulfate groups on the surface.^[^ ^28^ ^]^

**Figure 3:**
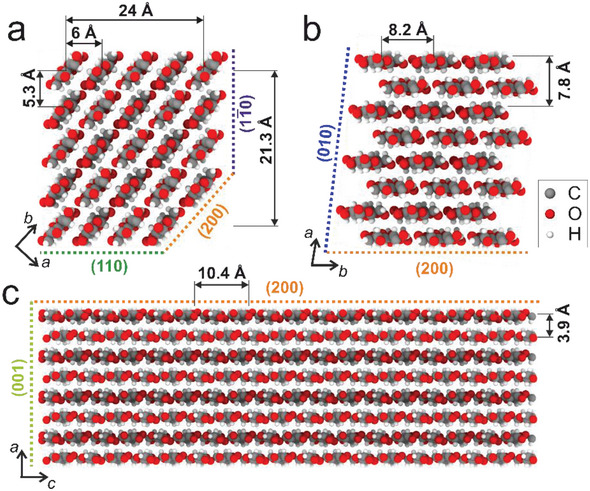
Main interchain distances observed in potential crystal habits of I_β_ elementary microfibrils comprised of 24 glucose chains with diamond a) and rectangular b) shape. c) Rectangular model with viewing direction parallel to the (200) plane.

Models for the cross‐sectional shape and size of individual wood CNCs are vividly discussed in the literature, while more recent studies favour 18 and 24‐chain models.^[^ ^9^, ^10^, ^11^, ^12^, ^29^, ^30^, ^31^, ^32^ ^]^ Two possible chain arrangements on 24 chain models with the corresponding characteristic distances are provided in **Figure** **3**. According to these models, a value of 3.9 Å is determined for the interlayer distance, while the distance between two C6 sites within the same chain is 10.4 Å, given by the periodicity of the anhydroglucose unit (AGU).^[^ ^5^ ^]^ It is to be expected that these distances are also reflected in the distances measured between individual Cs ions in our experiments. Assuming full occupation of all C6 sites at the CNCs surface, we would in principle expect to observe distances of 5.3, 6 or 8.2 Å between lines of Cs^+^, dependent on the face of the CNC oriented perpendicular to the viewing direction, corresponding to the periodicity of the crystal planes in the respective orientation. From this naive perspective, it seems unlikely that the (1$\bar{1}$0) or (200) planes are observed in Figure 2b.

**Figure 4:**
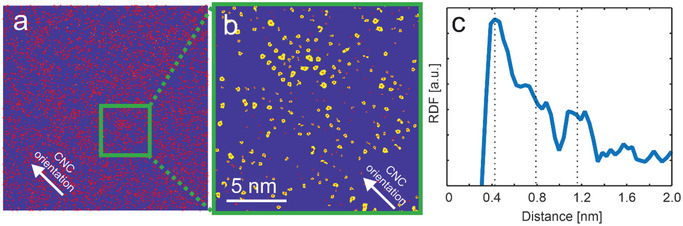
Determination of Cs^+^ atom positions from the HAADF image in Figure 2a. a) Overview of all determined atom positions. b) Magnified region highlighted in (a) showing the segmentation results with the centroid of each segment corresponding to the Cs atom positions. c) Radial density distribution (RDF) plot from all 6703 atom positions that were identified in (a).

To acquire more detailed information about interatomic distances on a larger scale, we conducted automatic segmentation of the HAADF image shown in Figure 2a using machine learning‐guided processing (described in the methods section).^[^ ^21^ ^]^ The segmentation results are given in yellow in **Figure** **4**a,b, and the obtained Cs positions, deduced from the centroids of each segmented region, are marked by red dots. It becomes even clearer from the segmented image that Cs^+^ are aligned along the *c*‐axis orientation of the CNCs in a regular pattern. A statistical analysis of the interatomic distances was performed on the obtained positions. A radial distribution function (RDF) was calculated from the 6703 identified atomic positions (Figure 3c) and reflects the observed ordering, however, with a relatively high variation in interatomic distances. A clear peak is discerned at ≈0.4 nm and can be assigned to the distance of Cs atoms within the same plane along the long axis, and corresponds well with the values measured in Figure 2c. Another broader peak is located between 0.6 and 1 nm and can be assigned to both a multiple of the first value (0.8 nm) and to the typically observed interline distance. Finally, a third peak is discerned between 1.0 and 1.3 nm, which corresponds reasonably well to the AGU periodicity and to a multiple of the first sharp peak. However, it is important to note that the RDF should be interpreted with caution, particularly at short distance values. The segmentation approach limits the resolution of closely spaced atoms, as two nearby Cs atoms are often identified as a single atom. This results in a rapid drop in the RDF at low distances.

For the interpretation of these experimental results, it is further pivotal to recognize that we observe the CNCs in projection. Given the limited depth of focus of the finely focused convergent electron beam (typically several nanometers^[^ ^33^ ^]^), it is impossible to discern whether a Cs atom is situated on the bottom or top of the CNC. This implies that the distances are measured between atoms attached to surfaces located at different heights of the CNCs. Consequently, the distance measurements are dependent on the crystallite's orientation with regard to the electron beam and tend to be lower than the actual 3D distances depicted in Figure 3, only reaching them in the case of precise parallel alignment. It should also be noted in this context that the underlying carbon support film, which is aligned perpendicular to the viewing direction, restricts the alignment of the CNCs^[^ ^34^ ^]^. While the CNCs rotation around the (001) axis in principle is arbitrary, its short axis rotation is determined by the flat support. As a consequence of the free orientation around (001), the parallel lines between Cs^+^ ions are only visible in regions where the CNCs are oriented accordingly with regard to the beam. Interestingly, we mostly do not see a higher density of Cs atoms at CNC border regions. Such arrangements would be expected to occur more frequently if the Cs atoms would be equally distributed over all CNC faces. This leads us to the conclusion that the sulfate groups must be attached to only a few preferred CNC facets. This conclusion also aligns with our observation that interatomic distances are much lower than to be expected for an equal distribution of the Cs atoms across all available surface sites, considering the 15 % coverage of CNC surfaces with sulfate groups (see the SI for details).

To verify the contrasts and distances observed in the STEM experiments, multislice simulations were conducted, considering potential locations of the Cs^+^ ions and thus the position of the sulfate groups.^[^ ^35^ ^]^ The C6 carbon positions of each surface AGU are considered the most likely bonding site for the sulfate group and hence the Cs^+^ location, since AGU hydroxyl group reactivity is typically defined to decrease in the order of C6, C2, and C3.^[^ ^36^ ^]^ As previously mentioned, the distances observed in projection depend on the presence of C6 bonding sites on the CNCs surfaces and on the geometric alignment of the surface facets with each other. Additionally, the C6 hydroxymethyl groups in cellulose can adopt three distinct conformations: trans‐gauche (tg), gauche‐trans (gt), and gauche‐gauche (gg). In bulk cellulose I, only the tg conformation is observed, where one of the C─O bonds adopts a trans orientation relative to the C5–C6 bond. This conformation is considered the most energetically favourable within the crystalline lattice due to hydrogen bonding stabilization^[^ ^5^ ^]^. At the surface, however, the absence of neighbouring chains to form hydrogen bonds alters the conformational landscape, making gg and gt conformations more likely. Indeed, both NMR experiments^[^ ^28^, ^37^ ^]^ and molecular dynamics simulations^[^ ^34^ ^]^ have shown that while all three conformations can be present, the gg conformation tends to dominate at the surface.

It is also important to note that the preferred conformation also strongly depends on the specific surface plane. For example, the (200) plane can form interchain hydrogen bonds, making the gg conformation energetically unfavourable. Similarly, on the (1$\bar{1}$0) plane, steric constraints restrict the accessibility of certain conformers. For both of these surfaces, the tg conformation was used instead in our structural models for the C6 bonding sites. For the simulations, we selected two different models representing diamond and rectangular cross‐sectional morphologies to encompass common surface facet configurations: (110) and (1$\bar{1}$0) or (200) and (010), respectively. Each model incorporates rigid S─O─Cs chains to approximate the sulfate groups at the surface, and each available C6 site on a specific surface is occupied. The hydrogen atoms are omitted in the whole geometry for simplicity, as their contribution to the HAADF contrast is negligible. To incorporate the contrast contribution from the amorphous carbon support to the HAADF signal, a randomly generated carbon support with a thickness of 2 nm was added to the model.^[^ ^38^ ^]^ Note that the model does not consider bond relaxation effects and interatomic interactions. A fixed value of 120° was chosen for the azimuthal angles of the bonds between O─S, S─O, and O─Cs, while the elevation angle is varied for each model in order to align the chains away from the CNCs surface.

**Figure 5:**
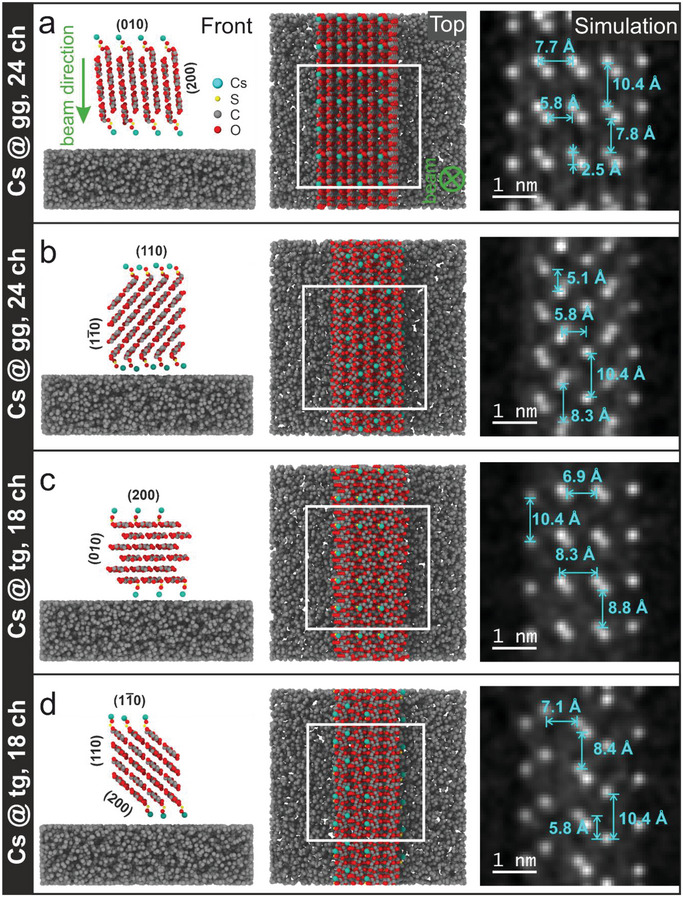
Modelling results based on 24 and 18 chain models with diamond shaped a,b) and rectangular c,d) cross‐sectional morphology, decorated with simplified sulfate groups at all surface C6 sites with gg conformation in (a,b) and C6 sites with the conformation in (c,d). For each geometry, the ball and stick model is given in front and top view, together with the multislice simulation result obtained from the area highlighted in the top‐view.

The simulation results are provided in **Figure** **5** together with the cross‐sectional and top views of the corresponding CNC model. For the examples shown, the CNCs were rotated in such a way to align one face decorated with sulfate groups perpendicular to the electron beam (and parallel to the carbon support). Other orientations are presented in the Supporting Information, demonstrating the effect of rotation around the [001]‐axis. From the simulated HAADF images, we can measure the distances between the bright spots representing the position of the Cs atoms, hence marking the position of the C6 bonding sites on the glucose chains. For each geometry and face, the most important distance values are provided. From these results, it can be concluded that the overall intensities and interatomic distances in the simulations are close to the values observed in the experiments, despite the simplicity of our model. For the distances between the chains, values between 5.8 and 8.3 Å are determined, which is within the range of the experimental values for all four models. However, the interatomic distances measured between the bright spots parallel to the chains (along the *c* or [001] axis) reveal some differences. For the rectangular model, all distances are either too low (below 3 Å) or too large (7.8 Å) in comparison with the typical experimental values (between 3 and 7 Å) for both faces (010) and (200). In contrast, for the diamond shaped model, lower values are measured that better align with the experimentally determined values in Figure 4 as well as in Figure 2.

Following our investigations, it is likely that the observed CNC structure exhibits a diamond‐shaped cross‐section rather than a rectangular one. The influence of CNC rotation on the measured projected distances is underscored by supplementary simulations detailed in the Supporting Information. Notably, the rotational flexibility of CNCs introduces complexity, rendering it challenging to unequivocally differentiate between the (011) and (0$\bar{1}$1) faces. However, the likelihood of the (200) face being present in the current scenario appears slim, primarily due to the discrepancies in measured distances and the number of parallel lines observed. In this context, the model's constraints suggest that only three rows of available C6 bonding sites support the structure observed, aligning with this assessment. It is worth noting, however, that in total only 15 % of C6 sites are actually occupied in the experiment, as we have estimated (see the SI),^[^ ^23^ ^]^ while in the simulations we assume full occupation of the surfaces observed. This might indicate that Cs is not homogeneously distributed, as for a homogeneous distribution of Cs^+^ over all available C6 sites, we would expect to measure larger distances between Cs^+^ occupied sites.

An intriguing aspect uncovered in our investigation relates to the relatively low levels of observed beam damage, allowing us to image regular patterns in many parts of the CNCs with HAADF. Notably, the critical dose for wood cellulose, indicative of the point at which diffraction spot intensity decreases by half, has been determined to be a mere 0.6 e‐/Å^2^ at room temperature with an electron energy of 200 keV.^[^ ^16^ ^]^ Radiolysis has been identified as the primary damage mechanism for organic solids such as cellulose, underscoring the efficacy of employing strategies like liquid nitrogen cooling and higher electron energies to mitigate beam‐induced damage.^[^ ^39^ ^]^ Balancing the signal‐to‐noise ratio with beam damage necessitates a judicious selection of the applied dose. It is important to acknowledge that our imaging parameters for capturing atomic resolution images significantly exceed the reported critical dose threshold. Nevertheless, our experiments reveal that the carbon substrate underlying the cellulose nanocrystals (CNCs), possessing good charge and heat conduction properties, exerts a significant stabilizing influence. In contrast, free‐standing CNCs on holey carbon supports demonstrated diminished stability, precluding successful imaging under our current experimental conditions. Furthermore, the distinctive bonding configuration of cellulose is believed to contribute significantly to its stability at the sub‐nanometer scale under electron beam exposure. The interplay of various forces within cellulose, encompassing van der Waals forces and hydrogen bonds between adjacent chains, alongside the presence of covalent glycosidic bonds linking anhydroglucose units along the chain, underscores the differential susceptibility of cellulose components to electron irradiation. This results in a rapid loss of crystalline order between chains upon exposure, leading to a swift decline in diffraction spot intensity, while internal chain order and sulfate groups likely exhibit a greater resilience to beam‐induced disruption, enduring for longer durations. Consequently, as the underlying structure degrades under electron beam irradiation, the atomic positions become increasingly randomized. The Cs atoms are stripped off of their bonding site and eventually tend to form small clusters with reduced interatomic distances.

## 2 Conclusion

The labeling of cellulose with cesium ions offers a powerful method for assessing structural arrangements within cellulose supramolecular structures at high resolution. Our study demonstrates the versatility of this technique, particularly when combined with atomically resolved cryogenic scanning transmission electron microscopy, which can be adapted to various cellulose and carbon‐rich substrates. Notably, this method shows strong potential for identifying the locations of sulfate groups, overcoming limitations faced by solid‐state NMR and other spatial mapping techniques.

Beyond sulfate localization, this labeling approach may also provide insights into interchain distances, substitution patterns, and the localization of functional groups. By further reducing the impact of beam damage and incorporating meticulous atomistic modelling, this approach lays the groundwork for future studies enhancing our understanding of chain arrangements within elementary fibrils and cellulose architecture more broadly. While the direct imaging of cellulose's molecular structure remains a significant challenge, our technique paves the way for advancing the structural analysis of cellulose and other polysaccharides, fostering deeper insights into their structural dynamics.

## 3 Experimental Section

### Materials

Cellulose nanocrystals were purchased from Celluforce (Canada). The diameter and the charge had been determined to be ≈4 nm and 0.2 mmol g^−1^, respectively, and the mean z‐average ≈97 nm.^[^ ^22^, ^23^, ^40^ ^]^ AFM imaging gives an indication that crystal lengths were around and above a hundred nanometers. They were labeled with cesium ions using our literature procedure.^[^ ^22^ ^]^ The CNCs were dispersed in to 2 wt.% concentration in ‐water (200 g in total quantity). For converting into the acidic form, the pH of the suspension was adjusted to 2 using HCl and maintained for 60 min. After that, the suspension was dialyzed for four days. The Cs form (Cs^+^‐CNC) was prepared by adjusting the suspensions to 0.01 m CsCl followed by adjusting the pH to 9 with NaOH and stirring for 60 min. The product was dialyzed for 4 days in water. After the dialysis, the concentrations of the suspensions were 1.90 wt.% (stock CNC), 0.95 wt.% H‐CNC, and 1.06 wt.% Cs^+^‐CNC.

### Elemental Composition Analysis and X‐Ray Diffraction Analysis

The X‐ray photoelectron spectroscopy (XPS) analysis was performed using a PHI 5000 VersaProbe III Scanning XPS Microprobe at an angle of 45°. A drop of the CNC suspensions was placed on a silicon wafer (Siegert Wafers, Germany), dried and subjected to the XPS analysis. Bruker D8 powder XRD was used for crystallinity analysis. See the Supplementary Information for more details.

### STEM Experiments

STEM characterization was performed on a probe‐corrected FEI Titan G2 60–300 microscope operated at 300 kV. The microscope was equipped with a high‐sensitivity Super‐X EDX detector. To reduce beam damage effects the experiments were performed at liquid nitrogen temperature using a cryo‐TEM holder (Gatan, Model 626). The probe current was set to ≈10 pA, and a pixel sampling time of 3 µs was chosen for imaging. Focusing was performed at lower magnification, higher scanning speeds, and lower image resolution in order to minimize the dose. Collection angles for ADF and HAADF imaging were 19.5–59 and 62.2–214 mrad, respectively. The convergence angle was 19.6 mrad. With these settings, a spatial resolution of less than 70 pm was achievable.

Sample preparation was done by conventional drop‐casting. The CNC suspension was diluted with deionized water (1:40) and treated for 5 min in an ultrasonic bath. A droplet of the diluted suspension was then cast on TEM grids which were covered with a 2–3 nm thick amorphous carbon film (TedPella Inc., Prod.No. 0 1824). After drying at room temperature, the samples were directly transferred to the TEM and cooled with LN_2_. Data acquisition and analysis were done in the Gatan Microscopy Suite (GMS), version 3.4. The distance values in Figure 2c were determined by fitting Gaussian functions to the peaks in Origin Pro 2020 (by Origin Lab). From these fits a maximum error of ± 0.03 nm has been estimated for the distance values. The distribution and interatomic distances of the Cs ions were automatically determined from the images by a supervised learning approach based on a Random Forest classifier as implemented Ilastik (version 1.3.3).^[^ ^41^ ^]^ Each individual pixel in the image was classified into two categories: it belongs to a Cs atom or belongs to the background (blue). To suppress the influence of noise, an averaging filter with a 3 × 3 kernel has been applied before classification. From the thereby obtained image segmentation data, the atomic positions were obtained by calculating the centroid of each detected segment belonging to a Cs atom. The statistical evaluation of the resulting Cs atom positions and calculation of the Radial Distribution Function (RDF) was performed by a script using Matlab (R2020a).

### Modelling

STEM image contrast was simulated using the Multislice method as implemented in the Dr. Probe software package (version 1.1), which was developed by Juri Barthel^[^ ^35^ ^]^. The multislice calculations were based on a CNC geometry decorated with a simplified model of the Cs^+^ containing sulfate half ester groups.^[^ ^5^ ^]^ The settings were selected to resemble the experimental parameters. The atomistic geometries were generated automatically by a Matlab script (R2020a) starting from a CNC‐supercell generated in VESTA.^[^ ^42^ ^]^ The surface O positions to attach the sulfate groups were selected by their coordination, assuming the carbon 6 position was most favorable.^[^ ^43^ ^]^ The sulfate group orientation was defined by predefined elevation and azimuthal angles with regard of the C─O bond at the C6 hydroxymethyl group. Depending on the surface to which the sulfate groups were applied the geometry was transformed to tg or gg conformation by rotation. At a distance of 145 pm a sulfate atom was placed and a Cs atom another 250 pm apart. These distances were estimated based on typical chemical bond lengths that were found in literature.^[^ ^44^ ^]^ This was repeated for each surface C6 position. Finally, the Cs^+^‐CNC structure was placed on a layer of amorphous carbon resembling the carbon TEM substrate, which was generated randomly on the fly.^[^ ^38^ ^]^ The number of substrate C atoms was calculated from a typical density of amorphous carbon of ≈2000 g cm^−^^3^. For model generation and file conversion, atomsk has been used.^[^ ^45^ ^]^

## Conflict of Interest

The authors declare no conflict of interest

## Supporting information



Supporting Information

## Data Availability

The data that support the findings of this study are available from the corresponding author upon reasonable request.

## References

[1] A. Khodayari , U. Hirn , S. Spirk , A. W. van Vuure , D. Seveno , Cellulose 2021, 28, 6007.

[2] E. Kontturi , et al., Angewandte Chemie (International ed. in English) 2016, 55, 14455. 27761976 10.1002/anie.201606626

[3] Y. Nishiyama , et al., Biomacromolecules 2003, 4, 1013. 12857086 10.1021/bm025772x

[4] Y. Nishiyama , J. Sugiyama , H. Chanzy , P. Langan , J. Am. Chem. Soc. 2003, 125, 14300. 14624578 10.1021/ja037055w

[5] Y. Nishiyama , P. Langan , H. Chanzy , J. Am. Chem. Soc. 2002, 124, 9074. 12149011 10.1021/ja0257319

[6] A. Endler , S. Persson , Mol. Plant 2011, 4, 199. 21307367 10.1093/mp/ssq079

[7] M. C. Jarvis , Plant Physiol. 2013, 163, 1485. 24296786 10.1104/pp.113.231092 PMC3850196

[8] D. P. Oehme , et al., Plant Physiol. 2015, 168, 3.25786828

[9] H.‐C. Tai , et al., Nature plants 2023, 9, 1154. 37349550 10.1038/s41477-023-01430-z

[10] A. Khodayari , et al., Carbohydr. Polym. 2024, 343, 122415. 39174111 10.1016/j.carbpol.2024.122415

[11] H.‐C. Tai , C.‐S. Tsao , J.‐H. Lin , Nature plants 2024, 10, 1067. 38849570 10.1038/s41477-024-01727-7

[12] P. A. Penttilä , A. Paajanen , Nature plants 2024, 10, 1064. 38769445 10.1038/s41477-024-01689-w

[13] D. P. Oehme , et al., Cellulose 2015, 22, 3501.

[14] J. D. Kubicki , et al., Sci. Rep. 2018, 8, 13983. 30228280 10.1038/s41598-018-32211-w PMC6143632

[15] A. A. Baker , W. Helbert , J. Sugiyama , M. J. Miles , New Insight into Cellulose Structure by Atomic Force Microscopy Shows the Ia Crystal Phase at Near‐Atomic Resolution, 79, pp. 1139–1145, 10.1016/S0006-3495(00)76367-3. PMC1301009 10920043

[16] Y. Ogawa , H. Chanzy , J.‐L. Putaux , Cellulose 2019, 26, 5.

[17] Y. Ogawa , J.‐L. Putaux , Front. Chem. 2022, 10, 835663. 35242740 10.3389/fchem.2022.835663 PMC8886399

[18] Y. Ogawa , J.‐L. Putaux , Cellulose 2019, 26, 17.

[19] K. L. Stinson‐Bagby , R. Roberts , E. J. Foster , Carbohydr. Polym. 2018, 186, 429. 29456006 10.1016/j.carbpol.2018.01.054

[20] R. Kádár , S. Spirk , T. Nypelö , ACS Nano 2021, 15, 7931. 33756078 10.1021/acsnano.0c09829 PMC8158857

[21] K. Heise , Adv. Mater. 2021, 33, 2004349. 33289188 10.1002/adma.202004349 PMC11468234

[22] P. Petschacher , R. Ghanbari , C. Sampl , H. Wiltsche , R. Kádár , S. Spirk , T. Nypelö , Nanomaterials 2022, 12, 3131. 36144921 10.3390/nano12183131 PMC9502719

[23] S. L. Navarro , et al., Cellulose 2021, 28, 9633.

[24] O. L. Krivanek , et al., Nature 2010, 464, 571. 20336141 10.1038/nature08879

[25] A. V. Crewe , J. Wall , J. Langmore , Science 1970, 168, 1338. 17731040 10.1126/science.168.3937.1338

[26] T. Furnival , et al., Appl. Phys. Lett 2018, 113, 183104.

[27] D. Knez , et al., Ultramicroscopy 2018, 192, 69. 29902687 10.1016/j.ultramic.2018.05.007

[28] A. N. Fernandes , L. H. Thomas , C. M. Altaner , P. Callow , V. T. Forsyth , D. C. Apperley , C. J. Kennedy , M. C. Jarvis , Proc. Natl. Acad. Sci. U.S.A. 2011, 108, E1195. 22065760 10.1073/pnas.1108942108 PMC3223458

[29] B. T. Nixon , et al., Sci. Rep. 2016, 6, 28696. 27345599 10.1038/srep28696 PMC4921827

[30] A. Zitting , A. Paajanen , P. A. Penttilä , Cellulose 2023, 30, 8107.

[31] M. C. Jarvis , Cellulose 2023, 30, 667.

[32] D. J. Cosgrove , Nature reviews. Molecular cell biology 2024, 25, 340. 38102449 10.1038/s41580-023-00691-y

[33] D. Knez , C. Gspan , N. Šimić , S. Mitsche , H. Fitzek , K. Gatterer , Commun. Mater. 2024, 5, 19.

[34] S. Fujisawa , K. Daicho , A. Yurtsever , T. Fukuma , T. Saito , Small 2023, 19, 2302276. 10.1002/smll.202302276 37183294

[35] J. Dr Barthel , Ultramicroscopy 2018, 193, 1. 29906518 10.1016/j.ultramic.2018.06.003

[36] D. Klemm , Fundamentals and analytical methods, Wiley‐VCH, Weinheim 1998.

[37] R. H. Newman , T. C. Davidson , Cellulose 2024, 11, 23.

[38] D. Knez , et al., Appl. Phys. Lett. 2019, 115, 123103.

[39] R. F. Egerton , Micron (Oxford, England : 1993) 2019, 119, 72. 30684768 10.1016/j.micron.2019.01.005

[40] S. L. Navarro , M. Tõlgo , L. Olsson , T. Nypelö , Cellulose 2023, 30, 9331.

[41] S. Berg , D. Kutra , T. Kroeger , C. N. Straehle , B. X. Kausler , C. Haubold , Nat. Methods 2019, 16, 1226. 31570887 10.1038/s41592-019-0582-9

[42] K. Momma , F. Izumi , J. Appl. Crystallogr. 2011, 44, 1272.

[43] S. Eyley , W. Thielemans , Nanoscale 2014, 6, 7764. 24937092 10.1039/c4nr01756k

[44] W. M. Haynes CRC Handbook of Chemistry and Physics, 95th ed., CRC Press, Hoboken 2014.

[45] P. Hirel , Comput. Phys. Commun. 2015, 197, 212.

