# Supplementary material for: Visualization of Cellulose Structures with Cesium Labeling and Cryo‐STEM
Source: Small. 2025 May 2;21(25):2500351. doi: 10.1002/smll.202500351 (PMC12199095; doi:10.1002/smll.202500351)
Supplement: Supplementary file 1 — Supporting Information [file SMLL-21-2500351-s001.pdf]

NANO · MICRO
small

Supporting Information

for *Small*, DOI 10.1002/smll.202500351

Visualization of Cellulose Structures with Cesium Labeling and Cryo-STEM

Daniel Knez, Patrick Petschacher, Helmar Wiltsche, Jean-Luc Putaux, Yoshiharu Nishiyama, Yu Ogawa, Gerald Kothleitner, Tiina Nypelö* and Stefan Spirk**

Supporting Information: Visualization of Cellulose Structures with Cesium Labeling and Cryo-STEM

Daniel Knez[1*], Patrick Petschacher[2], Helmar Wiltsche[3], Jean-Luc Putaux[4], Yoshiharu Nishiyama[4], Yu Ogawa[4], Gerald Kothleitner[1,5], Tiina Nypelö[6,7]*, Stefan Spirk[2]*

[1]Institute of Electron Microscopy and Nanoanalysis, Graz University of Technology, 8010 Graz, Austria

[2]Institute of Bioproducts and Paper Technology, Graz University of Technology, 8010 Graz, Austria

[3]Institute of Analytical Chemistry and Food Chemistry, Graz University of Technology, 8010 Graz, Austria

[4]Univ. Grenoble Alpes, CNRS, CERMAV, 38000 Grenoble, France

[5]Graz Center for Electron Microscopy, 8010 Graz, Austria

[6]Department of Chemistry and Chemical Engineering, Chalmers University of Technology, 41296 Gothenburg, Sweden

[7]Department of Bioproducts and Biosystems, Aalto University, 00076 Aalto, Finland

*corresponding author email: knez@tugraz.at, stefan.spirk@tugraz.at, tiina.nypelo@chalmers.se

Elemental composition

An amount of 0.32 at% of cesium was identified in the labeled CNCs (Cs-CNC) while the unlabeled grade (CNC) contained no cesium traces (Table S1). The sulfur content was 0.48 at% for the grade that was used for the visualization experiments. For four batches we report average sulfur and cesium contents of 0.47±0.04 and 0.31±0.10 at%.

Table S1. Atomic composition of CNC and Cs-CNC in at.%

	C1s	O1s	Na1s	S2p	Cs3d
**CNC**	57.93	41.18	0.54	0.35	--
**Cs-CNC**	58.19	41.01	0.48		0.32

We have previously reported the CNCs to contain approximately 0.74 wt% of sulfur based on elemental analysis[1]. The sulfate half ester groups are considered to be on CNC surfaces and the factual coverage of CNC surfaces was estimated in our previous work to be approximately 15%, based on similar elemental composition[2].

It should be noted, however, that while XPS is a highly surface-sensitive technique, it still exhibits a penetration depth of several nanometers. As a result, the detected signal includes a substantial contribution from the interior regions of the CNCs, which do not contain sulfate groups. Given that the surface represents only approximately 30% of the volume of an individual CNC, the measured sulfate content is effectively diluted by the sulfate-free core.

X-Ray diffraction analysis and crystallinity determination

XRD scan over 2θ 5-60° for CNC-Na and CNC-Cs exhibited same bands stemming from the semicrystalline order (Figure S1).

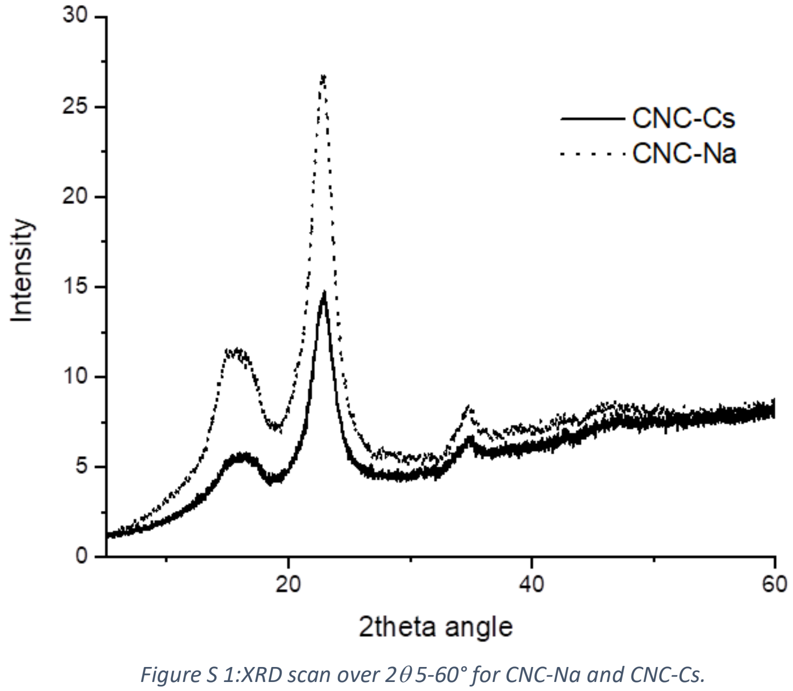

Figure S 1:XRD scan over 2θ 5-60° for CNC-Na and CNC-Cs.

Visualization of Cs arrangements and individual CNCs

To further visualize the linear arrangements of Cs and the individual CNCs we applied a Savitzky-Golay filter using a 11 pixel window, followed by a baseline subtraction using a rolling ball filter with a radius of 20 pixel to Figure 2a of the main manuscript. The result is shown in Figure S2 demonstrating the arrangment of Cs corresponding with the main orientation of the CNCs in the the image. Furthermore, also individual CNCs can be discerned clearly (CNC edges denoted by green dotted lines in the magnified region), each CNC is decorated by 3-4 lines of Cs atoms. For the CNC diameter a value of 3-3.2 nm has been measured from this region. Savitzky-Golay filtering was performed with an in-house C++ code and Roling-ball background subtraction was done using ImageJ (Fiji).

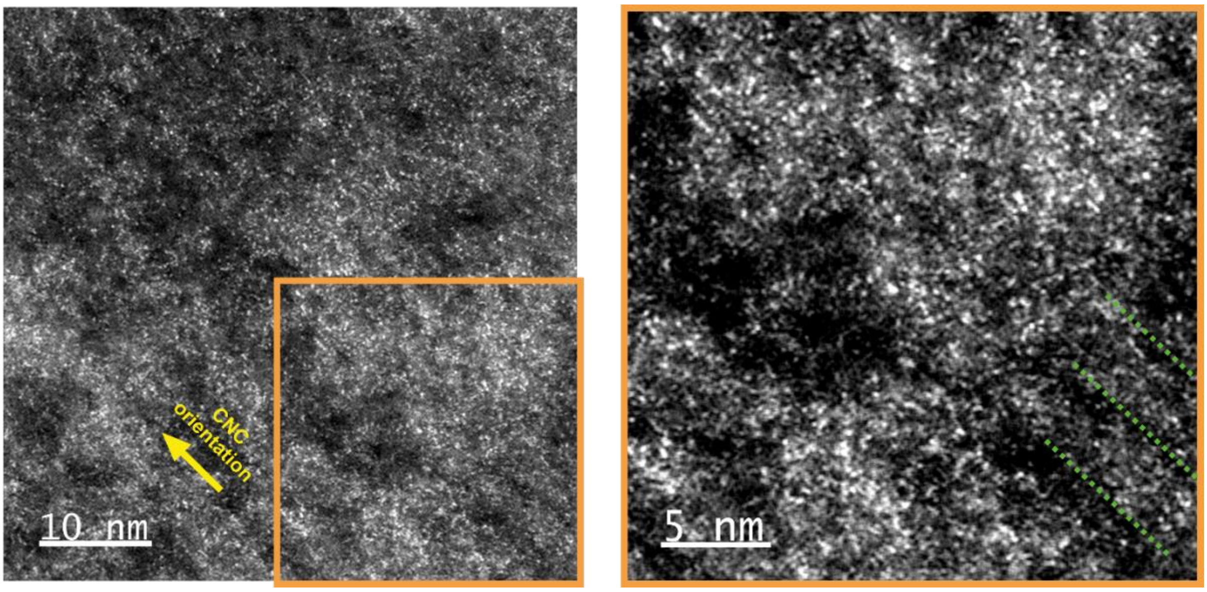

Figure S 2: (a) filtered HAADF image from the image provided in Fig2a of the main manuscript and a magnified region (denoted by the orange square), showing the linear arrangements of Cs atoms following the main orientation of the CNCs in the image and the extension of individual CNCs (CNC edges highlighted by green dotted lines). 3-4 lines of Cs are discernable on each CNC.

Additional HAADF images

Figure S3 presents additional images from four distinct sample regions, illustrating the typical alignment of Cs atoms along the principal orientation of the underlying CNCs. Figure S3a depicts an overlapping region where two CNC bundles intersect; as a result, the Cs atom alignment is disrupted, and no clear parallel lines are visible. In contrast, Figures S3b–d show regions where parallel lines can be observed, reflecting the underlying CNC order-however, the individual Cs atoms are not always resolved within these lines.

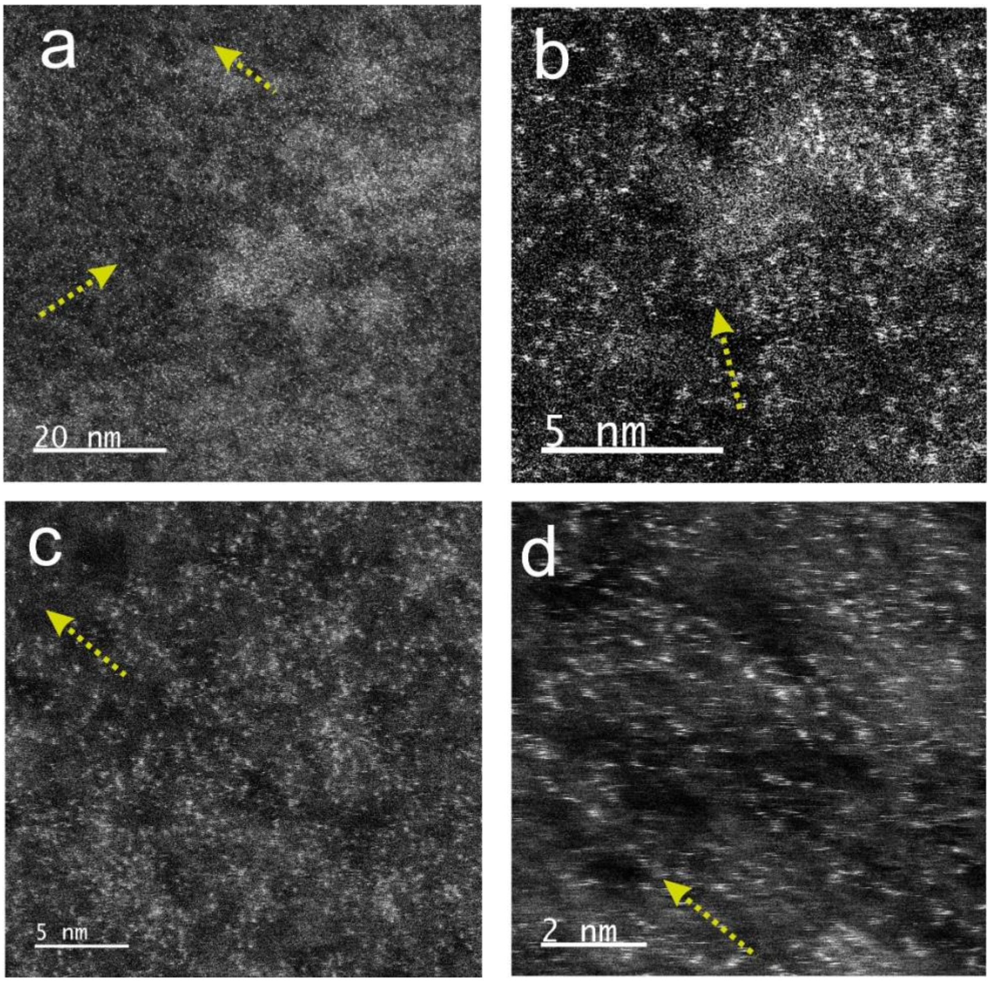

Figure S 3:Supplementary HAADF images from four different regions showing Cs ordering

Additional MS Simulations and the Influence of CNC rotation

As a supplement to the simulations presented in Fig. 5 in the main manuscript additional simulations are presented in Figure S 3. Figures in (a-b) show the influence of rotation on measured interatomic distances. The rotation angles were chosen in such a way to align the Cs ions attached at the bottom face with those at the top face to form lines along the [001] - axis, hence minimizing the distances measured along this crystallographic direction. Figure S 3c shows the same model with only the (200) face sulfated without rotation. (d- e) show two additional 24 chain model corresponding to the 18 chain models presented in Fig 5 of the main manuscript, for comparison.

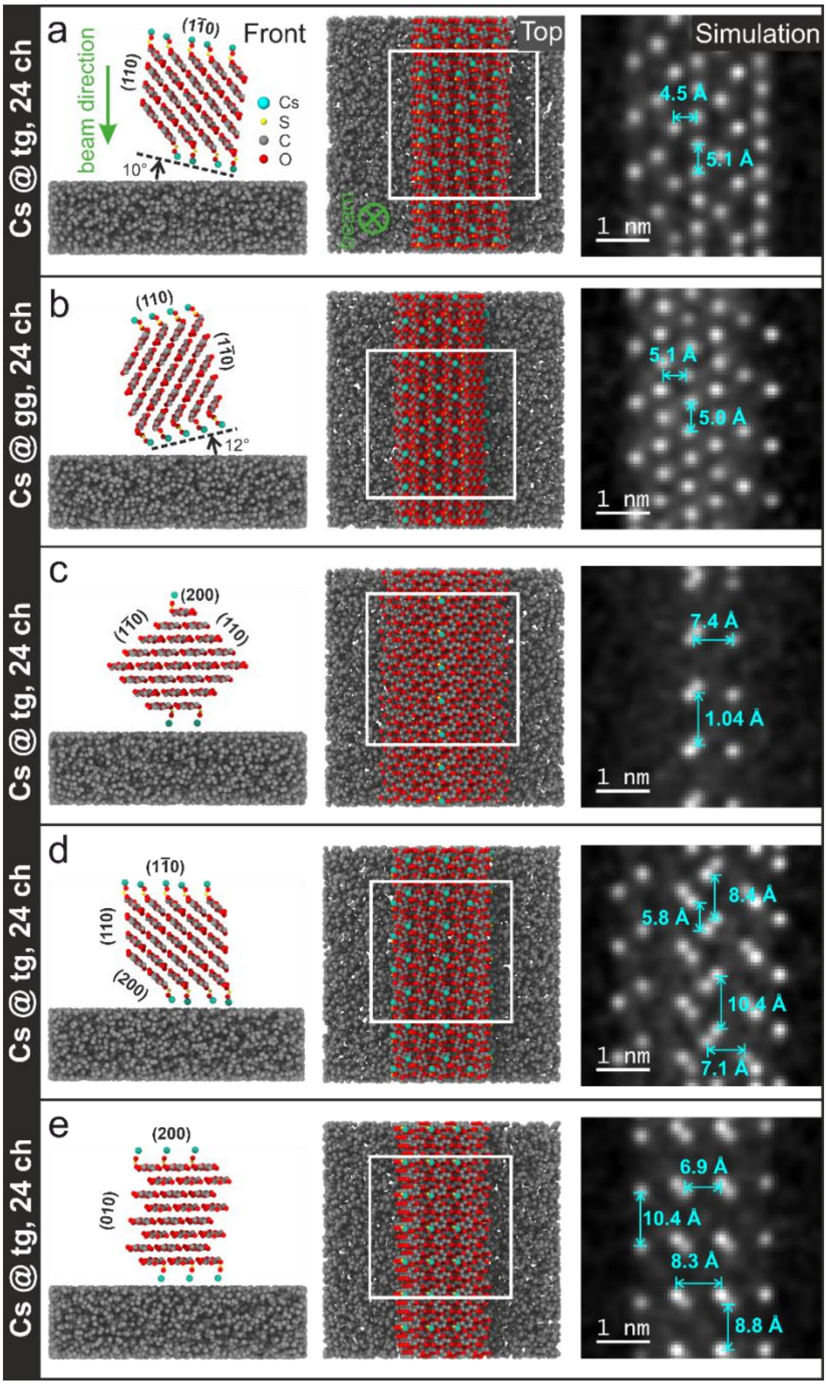

Figure S 4: Modelling results based on the 24 chain model with diamond shaped cross-sectional morphology, decorated with simplified sulfate groups at all surface C6 sites with gg (a) and tg (b) conformation with additional rotation along the (001) axis.

References

1. Petschacher, P. *et al.* Dynamic and Static Assembly of Sulfated Cellulose Nanocrystals with Alkali Metal Counter Cations. *Nanomaterials (Basel, Switzerland)* **12**; 10.3390/nano12183131 (2022).
2. Llàcer Navarro, S. *et al.* The effect of sulfate half-ester groups on cellulose nanocrystal periodate oxidation. *Cellulose* **28,** 9633–9644; 10.1007/s10570-021-04115-y (2021).
